# Current real-life use of vasopressors and inotropes in cardiogenic shock - adrenaline use is associated with excess organ injury and mortality

**DOI:** 10.1186/s13054-016-1387-1

**Published:** 2016-07-04

**Authors:** Tuukka Tarvasmäki, Johan Lassus, Marjut Varpula, Alessandro Sionis, Reijo Sund, Lars Køber, Jindrich Spinar, John Parissis, Marek Banaszewski, Jose Silva Cardoso, Valentina Carubelli, Salvatore Di Somma, Alexandre Mebazaa, Veli-Pekka Harjola, for the CardShock study investigators, Veli-Pekka Harjola, Marek Banaszewski, Lars Kober, Johan Lassus, Alexandre Mebazaa, Marco Metra, John Parissis, Jose Silva-Cardoso, Alessandro Sionis, Salvatore Di Somma, Jindrich Spinar, Katerina Koniari, Astrinos Voumvourakis, Apostolos Karavidas, Jordi Sans-Rosello, Montserrat Vila, Albert Duran-Cambra, Marco Metra, Michela Bulgari, Valentina Lazzarini, Jiri Parenica, Roman Stipal, Ondrej Ludka, Marie Palsuva, Eva Ganovska, Petr Kubena, Matias G. Lindholm, Christian Hassager, Tom Bäcklund, Raija Jurkko, Kristiina Järvinen, Tuomo Nieminen, Kari Pulkki, Leena Soininen, Reijo Sund, Ilkka Tierala, Jukka Tolonen, Marjut Varpula, Tuomas Korva, Anne Pitkälä, Rossella Marino, Alexandra Sousa, Carla Sousa, Mariana Paiva, Inês Rangel, Rui Almeida, Teresa Pinho, Maria Júlia Maciel, Janina Stepinska, Anna Skrobisz, Piotr Góral

**Affiliations:** Emergency Medicine, University of Helsinki and Department of Emergency Medicine and Services, Helsinki University Hospital, PO Box 340, 00029 HUS Helsinki, Finland; Division of Cardiology, Heart and Lung Center, University of Helsinki and Helsinki University Hospital, Helsinki, Finland; Intensive Cardiac Care Unit, Cardiology Department, Hospital de la Santa Creu i Sant Pau, Biomedical Research Institute Sant Pau (IIB Sant Pau), Barcelona, Spain; Department of Social Research, Faculty of Social Sciences, Centre for Research Methods, University of Helsinki, Helsinki, Finland; Rigshospitalet, Copenhagen University Hospital, Division of Heart Failure, Pulmonary Hypertension and Heart Transplantation, Copenhagen, Denmark; Department of Internal Medicine and Cardiology, University Hospital Brno, Brno, Czech Republic; Heart Failure Clinic and Secondary Cardiology Department, Attikon University Hospital, Athens, Greece; Institute of Cardiology, Intensive Cardiac Therapy Clinic, Warsaw, Poland; Department of Cardiology, University of Porto, CINTESIS, Porto Medical School, São João Hospital Center, Porto, Portugal; Division of Cardiology, Department of Medical and Surgical Specialties, Radiological Sciences, and Public Health, University and Civil Hospital of Brescia, Brescia, Italy; Department of Medical Sciences and Translational Medicine, University of Rome Sapienza, Emergency Medicine Sant’Andrea Hospital, Rome, Italy; INSERM U942, Hopital Lariboisiere, APHP and University Paris Diderot, Paris, France

**Keywords:** Cardiogenic shock, Vasoactive medication, Vasopressors, Inotropes, Adrenaline, Mortality, Survival, Propensity score

## Abstract

**Background:**

Vasopressors and inotropes remain a cornerstone in stabilization of the severely impaired hemodynamics and cardiac output in cardiogenic shock (CS). The aim of this study was to analyze current real-life use of these medications, and their impact on outcome and on changes in cardiac and renal biomarkers over time in CS.

**Methods:**

The multinational CardShock study prospectively enrolled 219 patients with CS. The use of vasopressors and inotropes was analyzed in relation to the primary outcome, i.e., 90-day mortality, with propensity score methods in 216 patients with follow-up data available. Changes in cardiac and renal biomarkers over time until 96 hours from baseline were analyzed with linear mixed modeling.

**Results:**

Patients were 67 (SD 12) years old, 26 % were women, and 28 % had been resuscitated from cardiac arrest prior to inclusion. On average, systolic blood pressure was 78 (14) and mean arterial pressure 57 (11) mmHg at detection of shock. 90-day mortality was 41 %. Vasopressors and/or inotropes were administered to 94 % of patients and initiated principally within the first 24 hours. Noradrenaline and adrenaline were given to 75 % and 21 % of patients, and 30 % received several vasopressors. In multivariable logistic regression, only adrenaline (21 %) was independently associated with increased 90-day mortality (OR 5.2, 95 % CI 1.88, 14.7, *p* = 0.002). The result was independent of prior cardiac arrest (39 % of patients treated with adrenaline), and the association remained in propensity-score-adjusted analysis among vasopressor-treated patients (OR 3.0, 95 % CI 1.3, 7.2, *p* = 0.013); this was further confirmed by propensity-score-matched analysis. Adrenaline was also associated, independent of prior cardiac arrest, with marked worsening of cardiac and renal biomarkers during the first days. Dobutamine and levosimendan were the most commonly used inotropes (49 % and 24 %). There were no differences in mortality, whether noradrenaline was combined with dobutamine or levosimendan.

**Conclusion:**

Among vasopressors and inotropes, adrenaline was independently associated with 90-day mortality in CS. Moreover, adrenaline use was associated with marked worsening in cardiac and renal biomarkers. The combined use of noradrenaline with either dobutamine or levosimendan appeared prognostically similar.

**Electronic supplementary material:**

The online version of this article (doi:10.1186/s13054-016-1387-1) contains supplementary material, which is available to authorized users.

## Background

Cardiogenic shock (CS) is a devastating complication of not only acute myocardial infarction (AMI) but also other cardiac emergencies [1]. While the prognosis of CS caused by AMI has improved, possibly due to increasing use of primary percutaneous coronary intervention (PCI) as early revascularization, mortality still remains unacceptably high [2, 3].

Cardiogenic shock is a result of severe impairment of cardiac output, and also of neurohormonal and cytokine activation, leading to systemic inflammatory response syndrome [1]. Fluid resuscitation, vasopressor and inotrope medications, and mechanical circulatory support have been used to stabilize severely impaired hemodynamics. However, the most commonly used form of mechanical support, the intra-aortic balloon pump (IABP) was not demonstrated to be of benefit in the recent IABP SHOCK II trial [4, 5]; nor have other devices yet been demonstrated to improve the prognosis of patients in CS [6]. Thus, use of vasopressors and inotropes remains a cornerstone of hemodynamic support in CS [7–10]. There are few data, however, to guide clinicians in the choice of specific agents. The overall benefit of vasoactive medications is unclear and safety concerns remain, as they may lead to increased myocardial oxygen and energy consumption. Moreover, they can have cardiotoxic effects and provoke arrhythmias [11–13], for instance, via beta-adrenergic stimulation resulting in excessive increase in free cytosolic Ca^2+^ and Ca^2+^ channel activation in cardiac myocytes.

We analyzed the current real-life use of vasoactive medications in a prospective observational study of patients with CS of various etiological causes. The associations between outcome and the use of vasopressors and inotropes were assessed to detect possible differences in safety profiles of vasopressor and inotropic therapy. We also assessed changes in hemodynamic parameters and biomarkers associated with the use of these medications.

## Methods

In the CardShock study (Clinicaltrials.gov identifier: NCT01374867) 219 patients with CS were prospectively enrolled at nine hospitals in eight European countries (Czech Republic, Denmark, Finland, Greece, Italy, Poland, Portugal, and Spain) in collaboration with the Global REsearch on Acute Conditions Team (GREAT) network. Recruitment started in October 2010 and ended on 31 December 2012 [14].

In addition to an acute cardiac cause, the inclusion criteria consisted of systolic blood pressure (SBP) <90 mmHg (in the absence of hypovolemia or after adequate fluid challenge) for at least 30 minutes, or need of vasopressor therapy to maintain adequate perfusion pressure, and signs of hypoperfusion (any of the following: altered mental status/confusion, cold periphery, oliguria, blood lactate >2 mmol/l). Patients had to be over 18 years of age and be included within 6 h of the first identification of shock. Informed consent was obtained from patients for study participation (apart from Copenhagen; see “Acknowledgements”). The main exclusion criteria were CS after cardiac surgery and ongoing hemodynamically significant arrhythmia as the cause of hypotension.

We recorded basic demographic data, previous medical history, and clinical, biochemical, and hemodynamic parameters on detection of shock and until 96 h after the study baseline. In addition cardiac index was registered in patients with a pulmonary artery catheter (n = 82 (38 %)). The etiology of CS was classified as acute coronary syndrome (ACS) or non-ACS; ACS was defined as ST elevation myocardial infarction (STEMI) or myocardial infarction without ST elevation (non-STEMI). Management of CS was registered in detail including intravenous medications, mechanical circulatory support, and ventilatory support. The use of vasoactive medications was recorded during the first 96 h after the detection of shock.

Noradrenaline, adrenaline, dopamine, vasopressin, and terlipressin were categorized as vasopressors, whereas dobutamine, levosimendan, and milrinone and enoximone (phosphodiesterase 3 inhibitors (PDE3i)) were categorized as inotropes. In addition to the use of each separate vasoactive drug, we analyzed the simultaneous use of different vasopressors and the combinations of vasopressors and inotropes. Serial blood samples were collected in 178 patients at 0 h, 12 h, 24 h, 48 h, 72 h and 96 h; plasma was immediately frozen and stored at −80 °C. Creatinine, high-sensitivity troponin T (hsTnT), and N-terminal pro-B-type natriuretic peptide (NT-proBNP) (Roche Diagnostics, Basel, Switzerland) were analyzed centrally from these samples. The endpoint of interest was 90-day all-cause mortality; three patients had missing follow-up data after hospital discharge. The CardShock study was approved by local ethics committees at the participating centers (see “Acknowledgements”) and conducted in accordance with the Declaration of Helsinki.

### Statistical analyses

Results are presented as number (*n*) and percentage (%) for categorical variables, and the mean with standard deviation (SD) or the median with interquartile range (IQR) for continuous variables, as appropriate. Group comparisons were performed using the Fisher exact or chi-square tests for categorical variables, and the *t* test or Mann-Whitney *U* test for continuous variables, as appropriate. Differences between groups over time in changes in biomarkers and hemodynamic parameters were tested with linear mixed modeling. Resuscitation-adjusted differences between groups at separate time points were assessed with linear regression. Due to skewed distribution the biomarkers were log-transformed to normalize the distribution and the residuals in these analyses. We performed multivariable logistic regression to evaluate independent associations between medications and mortality, adjusting for significant mortality-predicting variables included in the CardShock prediction model: age, previous myocardial infarction, previous coronary artery bypass graft (CABG), ACS as the etiological form of CS, left ventricular ejection fraction (LVEF), blood lactate, and confusion/altered mental status at baseline [14]; also gender and SBP were included in the model. Further adjustment included variables such as prior resuscitation (cardiac arrest), baseline creatinine, and IABP treatment.

To reduce bias and increase precision in analyses assessing the effect of treatment on mortality, we used propensity score adjustment and matching [15]. The variables chosen for propensity score analyses were potential confounders [16]; they were chosen based on clinical relevance and previous publications [5], giving priority, due to limited sample size, to variables believed or observed to be related to outcome [17], and on achieving balance between matched groups. The final propensity score was estimated with the following variables, also including strong predictors of outcome (i.e., the variables in the CardShock risk prediction model, as described previously): age, gender, medical history (myocardial infarction, CABG, hypertension, renal insufficiency), CS due to acute coronary syndrome, resuscitation prior to inclusion and initial presentation (confusion, blood lactate, creatinine, SBP, sinus rhythm, and LVEF). The score estimate was converted into a logit scale for propensity score adjustment analyses.

Propensity-score-matched subgroup analysis was performed both as sensitivity analysis and to corroborate the results from adjusted analyses of the impact of adrenaline on mortality. To maximize the sample size, patients with missing data were included, using the multiple imputation method with 3 imputations after 10 iterations; for LVEF the proportion of missing data was 5 %, and was 1 % or less for other variables used in matching. A 1:1 nearest neighbor match without replacement was used with a caliper <0.2 of the standard error of the logits of the propensity scores [18]. Balance between the matched groups was assessed as the standardized mean differences of the propensity scores and covariates used, and as the average of absolute standardized mean differences of the covariates.

We used the Kaplan-Meier method for unadjusted and Cox regression for adjusted survival analyses; the assumption of proportional hazards was checked with parallelism of log-log survival curves. Odds ratios (OR) and hazards ratios (HR) are shown with 95 % confidence intervals (95 % CI). We considered *p* values <0.05 as statistically significant. We performed statistical analyses with SPSS 23 statistical software (IBM Corp, Armonk, NY, USA). Additionally, IBM SPSS Statistics Essentials for R and SPSS PS Matching plugin [19] were used for propensity score matching.

## Results

Patient characteristics are shown in Table 1. A comprehensive description of the study population has been published previously [14]. Briefly, the mean age of patients was 67 (12) years and 26 % were women. On average, SBP was 78 (14) mmHg, mean arterial pressure 57 (11) mmHg, and heart rate 90 (28) beats/minute. LVEF was markedly reduced, 33 (14) %. ACS was the most common etiological cause of CS (81 % patients), and 84 % of these patients had STEMI. The characteristics of patients with CS of ACS and non-ACS etiology have been described recently [14]. Overall 90-day mortality was 41 %. Compared with survivors, non-survivors were older, more frequently had previous medical history of, for example, myocardial infarction, CABG, and renal insufficiency, and had lower LVEF, a worse biomarker profile, and less frequently sinus rhythm at baseline (Additional file 1). Furthermore, 60 patients (28 %) were resuscitated prior to inclusion.

**Table 1:** Patient characteristics in the overall population and in relation to adrenaline use

	All (n = 216)	Patients treated with adrenaline (n = 46)	Patients not treated with adrenaline (n = 170)	*P*
Age, mean (SD)	66.6 (11.8)	67.7 (9.4)	66.3 (12.4)	0.4
Women, %	26 %	28 %	25 %	0.7
Medical history, %				
Coronary artery disease	35 %	44 %	32 %	0.16
Previous MI	25 %	30 %	23 %	0.3
Previous PCI	15 %	15 %	14 %	0.9
Previous CABG	7 %	9 %	7 %	0.7
Heart failure	17 %	15 %	17 %	0.8
Hypertension	60 %	67 %	58 %	0.3
Diabetes	28 %	35 %	26 %	0.2
Renal insufficiency	12 %	20 %	9 %	0.051
Atrial fibrillation/flutter	15 %	17 %	14 %	0.5
Clinical presentation				
ACS etiology	81 %	87 %	79 %	0.2
Blood pressure, mmHg				
Systolic	78 (14)	77 (15)	78 (14)	0.9
Diastolic	47 (10)	44 (13)	47 (9)	0.1
Mean arterial pressure	57 (11)	55 (12)	57 (10)	0.3
Heart rate, beats/minute	90 (28)	96 (29)	89 (28)	0.15
Sinus rhythm	77 %	72 %	79 %	0.3
Clinical findings				
Cold periphery	95 %	98 %	94 %	0.3
Confusion	68 %	96 %	61 %	<0.001
Oliguria	57 %	82 %	49 %	<0.001
Lactate >2 mmol/l	72 %	93 %	66 %	<0.001
Resuscitation before enrolment	28 %	39 %	25 %	0.07
LVEF, %, mean (SD)	33 (14)	31 (15)	34 (14)	0.3
Biochemistry				
Hemoglobin, g/l	128 (22)	130 (22)	128 (22)	0.7
Creatinine, μmol/l	105 (80-139)	134 (91-171)	99 (80-135)	0.001
eGFR, ml/min/1.73 m^2^	60 (41–85)	45 (30–60)	64 (44–88)	<0.001
<60 ml/min/1.73 m^2^	50 %	76 %	43 %	<0.001
Lactate, mmol/l	2.9 (1.7–5.8)	5.6 (3.2–8.8)	2.5 (1.5–4.4)	<0.001
HsTnT, ng/l	2190 (393–5419)	2529 (463–4537)	2096 (357–6624)	0.8
NT-proBNP, ng/l	2710 (599–9583)	2088 (580–15753)	2995 (592–9133)	0.8

Results shown as % for categorical and mean (SD) or median (IQR) for continuous variables. *MI* myocardial infarction, *PCI* percutaneous coronary intervention, *CABG* coronary artery bypass graft surgery, *LVEF* left ventricular ejection fraction, *eGFR* estimated glomerular filtration rate (calculated using the Chronic Kidney Disease Epidemiology Collaboration equation), *hsTNT* high sensitivity troponin T, *NT-proBNP* N-terminal pro-B-type natriuretic peptide

### Use of vasopressors and inotropes

Vasopressors and inotropes were administered to 94 % of patients, and they were almost invariably initiated within the first 24 h (vasopressors in 98 % and inotropes in 94 % of patients). Noradrenaline (administered to 75 % of patients), dopamine (26 % of patients) and adrenaline (21 % of patients) were the most common vasopressors, while dobutamine (49 %) and levosimendan (24 %) were the most frequently used inotropes. Of note, a fair proportion of patients receiving adrenaline (39 %) were resuscitated from cardiac arrest prior to inclusion. The use of the medications, mortality effect, and maximum rates and duration of infusion are shown in Table 2. The use of vasopressors and inotropes was similar in patients with ACS and non-ACS.

**Table 2:** The use of vasoactive medications, their relationship with 90-day mortality, and maximum rates and duration of infusion

	Overall use *n* (%)	90-Day mortality				
		When treated with the respective agent	When treated without the respective agent	*P*	Maximum infusion rate, μg/kg/min, median (IQR)	Infusion duration, h
Vasopressors						
Noradrenaline	162 (75)	47 %	24 %	0.003	0.31 (0.16–0.63)	42 (20–72)
Adrenaline	46 (21)	74 %	32 %	<0.001	0.22 (0.10–0.36)	18 (6–41)
Dopamine	56 (26)	43 %	41 %	0.8	7.5 (3.2–11.1)	25 (8–71)
Vasopressin/terlipressin	8 (4)	88 %	39 %	0.01	NA	20 (9–29)
Inotropes						
Dobutamine	105 (49)	48 %	35 %	0.06	9.8 (6.5–16.7)	49 (16–72)
Levosimendan	52 (24)	33 %	44 %	0.15	0.13 (0.10–0.22)	40 (28–51)
PDE3i	9 (4)	33 %	42 %	0.6	NA	43 (21–72)
Combinations						
Vasopressor combination	65 (30)	66 %	30 %	<0.001	-	-
Dobutamine and vasopressor(s)	84 (39)	57 %	31 %	<0.001	-	-
Levosimendan and vasopressor(s)	47 (21)	34 %	44 %	0.3	-	-

*PDE3i* phosphodiesterase 3 inhibitor (milrinone or enoximone), NA, not applicable

Around half of the patients (n = 118; 55 %) received vasopressor-inotrope combinations, most often noradrenaline with either dobutamine (n = 81) or levosimendan (n = 47). The maximum noradrenaline dose was higher when used with dobutamine than with levosimendan (median 0.5 (IQR 0.30–0.99) vs 0.21 (0.12–0.38) μg/kg/min, *p* < 0.001).

A large proportion (29 %) of patients were treated exclusively with vasopressor(s) and one third of patients (n = 65) received more than one vasopressor (with or without concomitant inotropes). Of note, 10 % of patients received only inotropes.

**Fig. 1:**
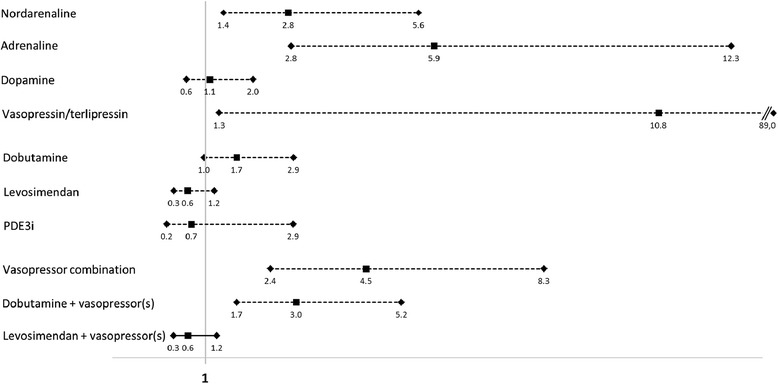
Risk of 90-day mortality according to vasopressor/inotrope medication: unadjusted odd ratios (squares) with 95 % confidence intervals. *PDE3i* phosphodiesterase 3 inhibitor (milrinone or enoximone)

In univariate regression analysis, the use of noradrenaline, adrenaline, vasopressin/terlipressin, vasopressor combination, or combination of dobutamine with vasopressor(s) but not levosimendan with vasopressor(s), were associated with increased 90-day mortality (Table 2, and Fig. 1).

### Adrenaline and 90-day mortality

The characteristics of patients who received or did not receive adrenaline are shown in Table 1. Additional file 2 shows a comparison within the subgroup of vasopressor-treated patients in relation to initial signs of hemodynamic stress and hypoperfusion, and to use of mechanical ventilatory and circulatory support.

A multivariable logistic regression model with variables included in the CardShock prediction model showed that only adrenaline was independently associated with increased 90-day mortality: OR 5.3, 95 % CI 1.88, 14.7, *p* = 0.002. Further adjustment with prior resuscitation, renal function or IABP treatment did not change the results in the model. The association remained similar even after propensity score adjustment, which was used to balance differences and covariates in patients who received or did not receive adrenaline: OR 3.3, 95 % CI 1.4, 7.7, *p* = 0.006.

**Fig. 2:**
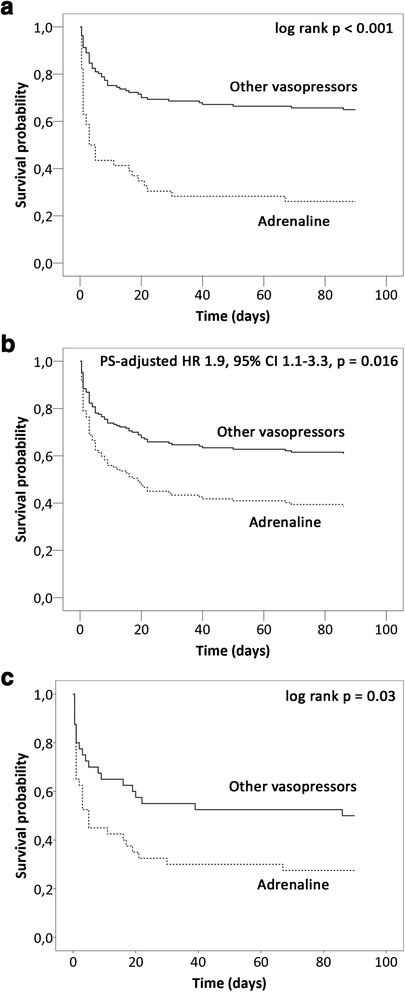
Survival curves for use of adrenaline (*dashed line*) vs other vasopressors (*solid line*). **a** unadjusted (Kaplan-Meier). **b** Propensity-score-adjusted (Cox regression, see below) *HR* hazard ratio. **c** Propensity-score-matched (one of the imputed cohorts; log rank *p* < 0.05 for all). The propensity score (*PS*) was estimated with the following variables: age, gender, medical history (myocardial infarction, coronary artery bypass graft surgery, hypertension, renal insufficiency), acute coronary syndrome as the etiology of cardiogenic shock, resuscitation prior to inclusion and initial presentation (confusion, blood lactate, creatinine, systolic blood pressure, sinus rhythm, and left ventricular ejection fraction). The score was converted to the logit scale for adjustment in Cox regression (**b**)

Kaplan-Meier analyses confirmed the association between adrenaline and increased 90-day mortality within this subgroup (Fig. 2a). The association remained consistent even after propensity score adjustment (Fig. 2b and Additional file 3). A subgroup analysis with propensity score matching in the three imputed cohorts produced a pool of 40 matched pairs. As Additional file 4 shows, no marked imbalances remained in the propensity score, the covariates used, or the average of the absolute standardized mean differences of the covariates (Additional file 4). On analysis of 90-day mortality in the three matched cohorts, the pooled OR for adrenaline was 2.8 (95 % CI 1.1, 7.1, *p* = 0.03). The Kaplan-Meier survival curve for a matched cohort is shown in Fig. 2c.

**Fig. 3:**
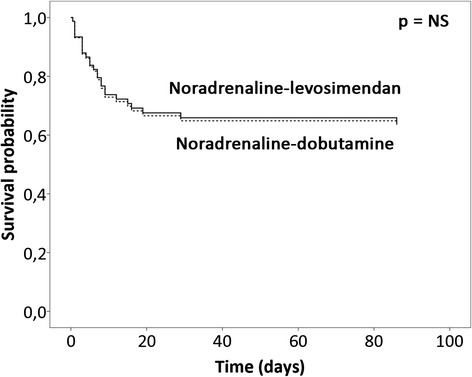
Survival-probability curves for propensity-score-adjusted Cox regression analysis for use of dobutamine (*dashed line*) and levosimendan (*solid line*) with noradrenaline. Adjusted for logit of the propensity score, which was estimated with the following variables: age, gender, medical history (myocardial infarction, coronary artery bypass graft surgery, hypertension, renal insufficiency), CS of acute coronary syndrome etiology, resuscitation prior to inclusion and initial presentation (confusion, blood lactate, creatinine, systolic blood pressure, sinus rhythm, and left ventricular ejection fraction). Of note, patients who received both dobutamine and levosimendan, or adrenaline were excluded. *NS* not significant

By contrast, Fig. 3 shows that after propensity score adjustment the two most frequent vasopressor-inotrope combinations, namely noradrenaline with either levosimendan or dobutamine, were associated with similar 90-day mortality (OR for noradrenaline-dobutamine = 1.03, 95 % CI 0.3, 3.9, *p* = 0.97).

### Impact of adrenaline on organ function

**Fig. 4:**
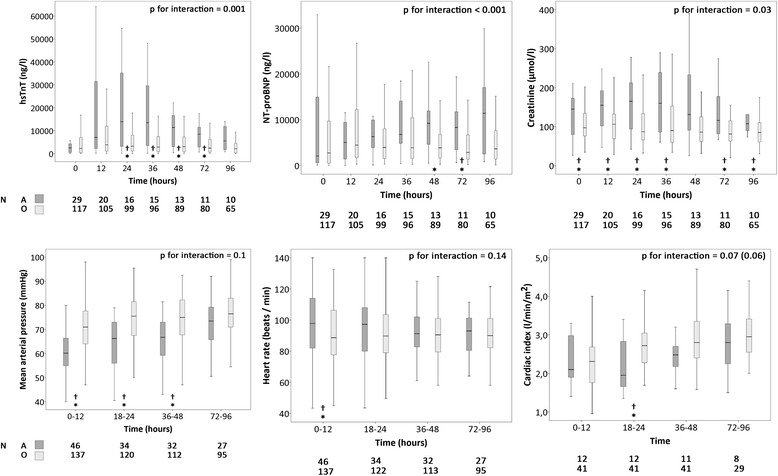
Hemodynamics and plasma biomarkers in patients receiving adrenaline (*dark grey*; *A*) or other vasopressor(s) (*light grey*; *O*). Boxplots show separate measurements in each time point in the *upper row* (biomarkers) and the mean values of time intervals in the *lower row* (hemodynamics): *central lines* median, *box*es interquartile range, *whiskers* minimum and maximum with outliers excluded, *A* adrenaline, *O* other vasopressor, *hsTnT* high sensitivity troponin T, *NT-proBNP* N-terminal pro-B-type natriuretic peptide. *P < 0.05 for difference between adrenaline and other vasopressors. †*P* < 0.05 for difference between adrenaline and other vasopressors, when adjusted for resuscitation. *P* value for time-by-group interaction (with or without adjustment for resuscitation) shown for biomarkers, mean arterial pressure and heart rate; for CI, *p* value with adjustment for resuscitation shown in *brackets*

When comparing adrenaline with other vasopressors (mainly noradrenaline and dopamine), the cardiac index and blood pressure were similar by 96 h in both subgroups (Fig. 4). However, cardiovascular and renal biomarker levels were much worse in patients who received adrenaline, with a more pronounced increase compared to other vasopressors over the initial 96 h after detection of shock (Fig. 4). When the analyses were repeated with adjustment for resuscitation before enrollment the differences remained significant (Fig. 4). Furthermore, subgroup analysis of non-resuscitated patients showed similar detrimental effects of adrenaline on organ function (Additional file 5). Patients treated with adrenaline more often had altered mental status (Additional file 2), and received higher maximum infusion rates of concomitant noradrenaline, or dobutamine (Additional file 6). In addition, lactate levels were higher in patients receiving adrenaline, with a 4-day median of 3.7 (IQR 1.8–7.1) mmol/l vs 1.3 (IQR 0.9–1.8) mmol/l in patients receiving other vasopressors.

By contrast, biomarker measurements did not demonstrate any clinically relevant differences between dobutamine and levosimendan when combined with noradrenaline, and the improvement in hemodynamics was similar in the two groups (Additional file 7).

## Discussion

This study describes the contemporary use of vasoactive medications and their association with 90-day survival in an unselected patient population with CS. Combined use of vasopressors and inotropes was common, mostly noradrenaline with either dobutamine or levosimendan. Adrenaline, regardless of the maximum infusion rate, was consistently associated with worse outcome. In addition, despite reaching similar hemodynamic stabilization as in patients treated with other vasopressors, patients treated with adrenaline had significant worsening in cardiac and renal biomarker profiles. We observed no difference in 90-day mortality between patients who received combination of dobutamine and noradrenaline and those who received levosimendan in combination with noradrenaline.

In light of previous data suggesting that adrenaline is less safe and has more side effects than other vasoactive medications, the use of adrenaline in the present study was unexpectedly frequent [13, 20, 21]. While adrenaline is recommended for resuscitation during cardiac arrest, most patients treated with adrenaline in the CardShock study, however, had not been resuscitated. More importantly, our study shows that adrenaline is associated with 90-day mortality independent of prior cardiac arrest, and even after further multivariable adjustment or using propensity score methods. Furthermore, compared with use of other vasopressors, adrenaline is associated with marked aggravation of cardiac stress, myocardial injury and kidney dysfunction during the 4 days following detection of shock. Strikingly, these associations remained consistent in the subgroup of patients with no prior cardiac arrest. This may reflect an increase in myocardial oxygen consumption, excessive vasoconstriction and/or direct organ (cardiac, kidney, or other) toxic effects due to intense adrenergic stimulation [22, 23]. Based on these findings, alternative treatment strategies seem preferable. These would include the use of other vasopressors and/or inotropes, or more advanced therapy, such as mechanical circulatory support, in CS patients requiring potent hemodynamic stabilization. The benefit of such strategies also needs to be promptly and properly investigated.

Noradrenaline was the most commonly used vasopressor; a finding in line with the current recommendations [7, 8, 22, 24]. Dopamine was also given to one fourth of patients. Although commonly classified as a vasopressor [8, 22, 25], most patients actually received it at low-intermediate, or “renal-inotropic” doses [26]. However, the drug has failed to show beneficial effects on outcome [27, 28]. Moreover, compared to noradrenaline it has a weaker vasopressor effect and more adverse effects [29, 30]. A randomized study comparing dopamine with noradrenaline in shock showed that arrhythmia was more frequent in the dopamine group, the drug was discontinued more often due to severe arrhythmia, and the outcome was worse in the subgroup with CS [11]. As the present study was observational, the management of patients was at the discretion of the local physicians in charge. Lack of randomized data and evidence on the benefits of different vasoactive medications, and concern about possible adverse effects make the choice between different agents challenging [20, 31]. In addition, local practices in the choice of vasoactive medication may vary, as has been reported in studies of acute heart failure [32, 33].

The paucity of scientific data on the use of inotropes and their effect on mortality in AMI complicated by CS is highlighted by a recent Cochrane review [34]. While vasopressors are indicated for correcting low perfusion pressure, inotropes are used to increase cardiac output in CS. A large retrospective analysis of three cohorts with acute heart failure suggested that combining a vasopressor with an inodilator (i.e., dobutamine, levosimendan, or PDE3i) in CS was associated with lower mortality compared to vasopressors alone [35]. In turn, our study showed that the combination of noradrenaline with levosimendan was not associated with excessive mortality. Then again, while the combination of dobutamine and noradrenaline was associated with increased mortality in unadjusted analysis, the adjusted analyses showed a similar outcome for the combination of noradrenaline with either dobutamine or levosimendan. More importantly, both achieved similar hemodynamic stabilization with no clinically relevant differences in serial cardiac or renal biomarkers. Altogether, levosimendan and dobutamine appeared to be equally useful alternatives to be combined with noradrenaline in the setting of unselected patients with CS. Considering the deleterious outcome related to adrenaline, a combination of dobutamine or levosimendan with noradrenaline could be the preferred choice in patients needing inotropic support.

There are some limitations to be acknowledged. First, there was no formal standardization of management in the CardShock study. However, the primary goal was to describe the current use of vasopressors and inotropes in CS and data on vasoactive treatments were prospectively collected. Second, the total dose of vasoactive medications, and duration of the maximum dose might have given further depth to the interpretation of data. However, these details were not registered. Third, the numbers of patients in the treatment groups including adrenaline or levosimendan were limited, and caution in the interpretation of the results is advocated. As the study lacks randomization, confounding by indication is a possible bias when assessing possible effect of adrenaline on mortality. Propensity score methods were used to minimize this bias; however, these methods allowed us only to account for the measured variables and the estimates of treatment effect may be susceptible to bias due to unknown and unmeasured confounding variables. Nevertheless, the association between adrenaline use and poor outcome seemed consistent. Finally, we classified dopamine as a vasopressor, although actual doses used and combining with other vasopressors might suggest a pursuit of “renal-preserving” or inotropic effect.

## Conclusions

Vasopressors and/or inotropes are almost invariably used in the treatment of CS and were initiated soon after detection of shock. Adrenaline, used alone or in combination with other vasoactive medication, was associated with worsening of cardiac and renal injury and increased mortality, raising questions about the safety of this treatment. In turn, levosimendan in combination with noradrenaline, was associated with a more positive outcome, and dobutamine with noradrenaline appeared to be a prognostically equal alternative; these combinations should be favored in the early management of CS. Because our study does not prove causality, our findings underscore the need for randomized controlled trials of adrenaline versus noradrenaline in CS.

## Key messages

- Adrenaline use in cardiogenic shock is independently associated with excess 90-day mortality
- Adrenaline use is associated with marked aggravation of cardiac stress, myocardial injury and kidney dysfunction
- Combined use of either dobutamine or levosimendan with noradrenaline is prognostically similar

## Abbreviations

ACS, acute coronary syndrome, AMI, acute myocardial infarction, CABG, coronary artery bypass graft; CS, cardiogenic shock, hsTnT, high-sensitivity troponin T, IABP, intra-aortic balloon pump, LVEF, left ventricular ejection fraction, non-STEMI myocardial infarction without ST elevation; NT-proBNP, N-terminal pro-B-type natriuretic peptide, PCI, percutaneous coronary intervention, PDE3i, phosphodiesterase 3 inhibitor, SBP, systolic blood pressure; STEMI, ST elevation myocardial infarction

## Additional files

Additional file 1: Table S1. Comparison of patient characteristics in survivors and non-survivors at 90 days. (PDF 33 kb) Additional file 2: Table S2. Comparison of initial signs and markers of hemodynamic stress, and use of invasive ventilatory and mechanical support in adrenaline-treated patients and those treated with other vasopressors. (PDF 19 kb) Additional file 3: Figure S1. Propensity-score-adjusted association between vasopressors and 90-day mortality among vasopressor-treated patients. (PDF 22 kb) Additional file 4: Table S3. Baseline characteristics used in propensity score matching in relation to 90-day mortality in patients treated with vasopressors, and the balance achieved between the propensity-score-matched groups. (PDF 28 kb) Additional file 5: Figure S2. Hemodynamics and plasma biomarkers in non-resuscitated patients receiving adrenaline or other vasopressor(s). (PDF 476 kb) Additional file 6: Table S4. Maximum infusion rates of vasoactive medications in patients treated with and without adrenaline. (PDF 8 kb) Additional file 7: Figure S3. Hemodynamics and biomarkers in patients receiving either a combination of noradrenaline and dobutamine or noradrenaline and levosimendan. (PDF 417 kb)
