# Supplementary material for: Current real-life use of vasopressors and inotropes in cardiogenic shock - adrenaline use is associated with excess organ injury and mortality
Source: Crit Care. 2016 Jul 4;20:208. doi: 10.1186/s13054-016-1387-1 (PMC4931696; doi:10.1186/s13054-016-1387-1)
Supplement: Additional file 1: Table S1. — Comparison of patient characteristics in survivors and non-survivors at 90 days. (PDF 33 kb) [file 13054_2016_1387_MOESM1_ESM.pdf]

Table S1. Comparison of patient characteristics between survivors and non-survivors at 90 days.

	**All** n=216	**Alive at 90 days** n=127	**Dead at 90 days** n=89	**p**
Age (mean; SD)	66.6 (11.8)	64.3 (12.2)	70.0 (10.5)	<0.001
Women (%)	26%	22%	33%	0.08
**Medical history (%)**				
Coronary artery disease	35%	24%	51%	<0.001
Previous MI	25%	16%	38%	<0.001
Previous PCI	15%	12%	19 %	0.14
Previous CABG	7%	2%	16%	<0.001
Heart failure	17%	13%	21%	0.12
Hypertension	60%	55%	67%	0.07
Diabetes	28%	22%	36%	0.03
Renal insufficiency	12%	19%	6%	0.004
Atrial fibrillation/flutter	15%	14%	16%	0.8

**Clinical presentation**				
ACS etiology	81%	77%	85%	0.1
Blood pressure (mmHg)				
Systolic	78 (14)	80 (14)	74 (12)	0.001
Diastolic	47 (10)	48 (9)	45 (11)	0.04
Mean arterial pressure	57 (11)	58 (10)	55 (11)	0.02
Heart rate (beats/min)	90 (28)	90 (26)	91 (31)	0.7
Sinus rhythm	77%	84%	67%	0.004
**Clinical findings**				
Cold periphery	95%	93%	98%	0.1
Confusion	68%	58%	83%	<0.001
Oliguria	57%	44%	75%	<0.001
Lactate >2 mmol/l	72%	58%	92%	<0.001
Resuscitation before enrolment	28%	23%	35%	0.05
LVEF [%, mean(SD)]	33 (14)	37 (15)	28 (12)	<0.001

**Biochemistry**				
Hemoglobin (g/l)	128 (22)	131 (21)	125 (23)	0.05
Creatinine (umol/l)	105 (80-139)	96 (73-127)	123 (91-174)	<0.001
eGFR (ml/min/1.73m^2)	60 (41-85)	67 (49-93)	45 (30-67)	<0.001
<60 ml/min/1.73m^2	50%	38%	66%	<0.001
Lactate (mmol/l)	2.9 (1.7-5.8)	2.2 (1.4-3.8)	5.0 (2.8-8.3)	<0.001
hsTnT (ng/l)	2190 (393-5419)	1390 (208-4191)	2862 (1050-8346)	0.009
NT-proBNP	2710 (599-9583)	2026 (469-7066)	5174 (1186-16549)	0.002

Results shown as % for categorical and mean (SD) or median (IQR) for continuous variables.

MI = myocardial infarction, PCI = percutaneous coronary intervention, CABG = coronary artery bypass graft surgery, LVEF = left ventricular ejection fraction, eGFR = estimated glomerular filtration rate (calculated using CKD-EPI equation), hsTNT = high sensitivity troponin T, NT-proBNP = N-terminal pro-B-type natriuretic peptide
