# Supplementary material for: Current real-life use of vasopressors and inotropes in cardiogenic shock - adrenaline use is associated with excess organ injury and mortality
Source: Crit Care. 2016 Jul 4;20:208. doi: 10.1186/s13054-016-1387-1 (PMC4931696; doi:10.1186/s13054-016-1387-1)
Supplement: Additional file 2: Table S2. — Comparison of initial signs and markers of hemodynamic stress, and use of invasive ventilatory and mechanical support in adrenaline-treated patients and those treated with other vasopressors. (PDF 19 kb) [file 13054_2016_1387_MOESM2_ESM.pdf]

Table S2. Comparison between adrenaline-treated patients and those treated with other vasopressors in relation to initial signs and markers of hemodynamic stress, as well as use of invasive ventilatory support, and mechanical support.

	**Adrenaline (n=46)**	**Other vasopressor (n=137)**	**p**
ACS etiology	87%	81%	0.4
STEMI (% of ACS)	78%	85%	0.3
NSTEMI (% of ACS)	23%	13%	0.2
PCI (% of ACS)	80%	87%	0.3
Resuscitation prior to enrolment	39%	27%	0.1
Clinical signs of hypoperfusion			
Cold periphery	98%	93%	0.3
Confusion	96%	61%	<0.001
Oliguria	82%	51%	<0.001
Lactate > 2 mmol/l	93%	65%	<0.001
Blood pressure (mmHg; mean (SD))			
systolic	77 (15)	78 (14)	0.9
diastolic	44 (13)	47 (10)	0.1
mean arterial pressure	55 (12)	57 (10)	0.4
Heart rate (bpm; mean (SD))	96 (29)	88 (27)	0.1
Sinus rhythm	72%	80%	0.2
LVEF (%)	31%	33%	0.5
Cardiac index (l/min/m^2; median (IQR))	2.1 (1.8-2.8)	2.0 (1.6-2.9)	0.5
Lactate (mmol/l; median (IQR))			
at inclusion	5.6 (3.2-8.6)	2.6 (1.5-4.9)	<0.001
at 24 hours	2.6 (1.6-6.7)	1.3 (1.0-1.8)	<0.001
**Mechanical support**			
Invasive ventilatory support	89%	60%	<0.001
Intra-aortic balloon pump	74%	56%	0.03
LVAD or ECMO	15%	2%	<0.001

SD = standard deviation, LVEF = left ventricular ejection fraction, IQR = interquartile range, ACS = acute coronary syndrome, PCI = percutaneous coronary intervention, LVAD = left ventricular assist device, ECMO = extracorporeal membrane oxygenation
