# Supplementary material for: Current real-life use of vasopressors and inotropes in cardiogenic shock - adrenaline use is associated with excess organ injury and mortality
Source: Crit Care. 2016 Jul 4;20:208. doi: 10.1186/s13054-016-1387-1 (PMC4931696; doi:10.1186/s13054-016-1387-1)
Supplement: Additional file 3: Figure S1. — Propensity-score-adjusted association between vasopressors and 90-day mortality among vasopressor-treated patients. (PDF 22 kb) [file 13054_2016_1387_MOESM3_ESM.pdf]

Figure S1. Propensity score adjusted association of vasopressors with 90-day mortality among vasopressor-treated patients (n=183). Figure represents ORs with 95% CI.

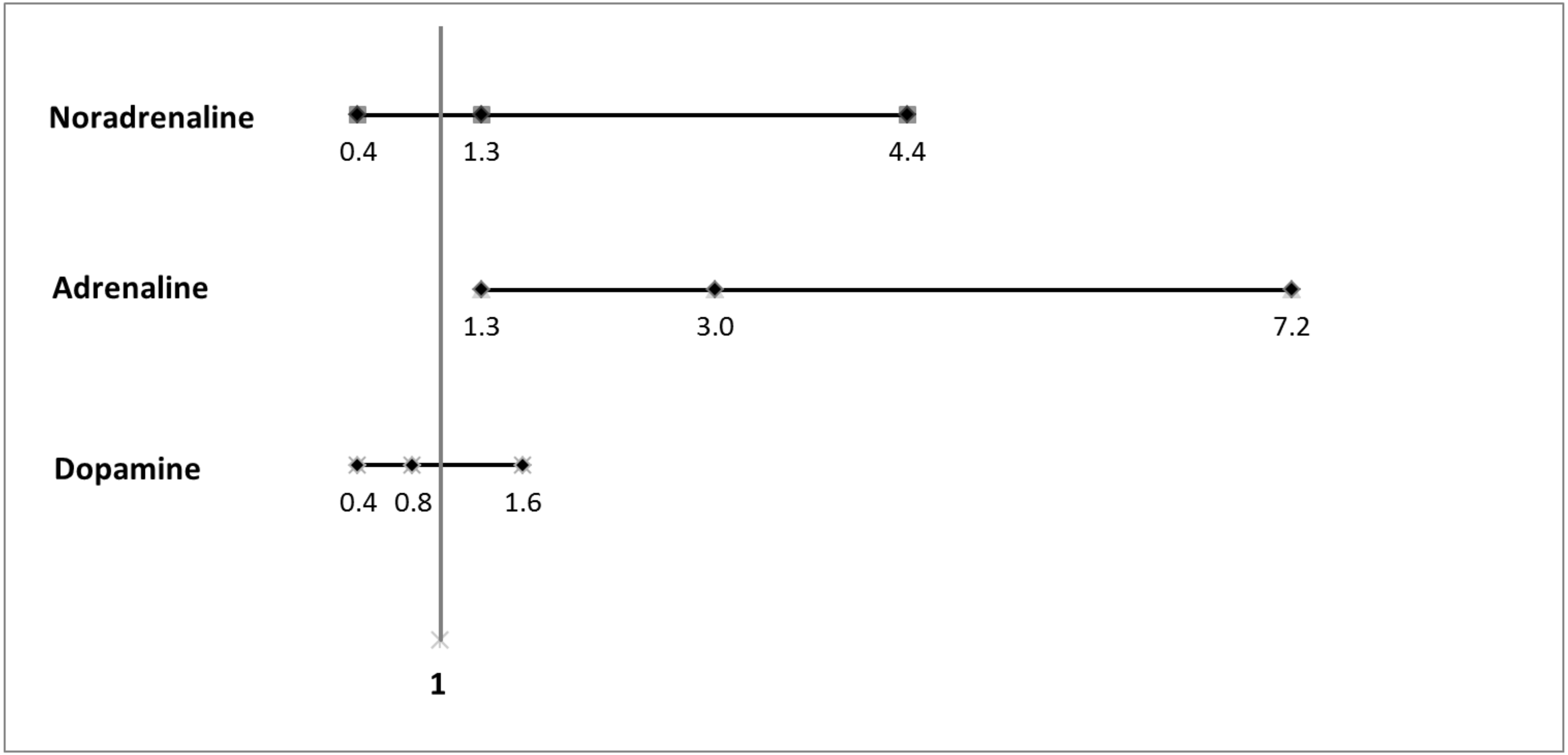

Adjusted for logit of the propensity score, which was estimated with the following variables: age, gender, medical history (myocardial infarction, coronary artery bypass graft surgery, hypertension, renal insufficiency), acute coronary syndrome as CS etiology, and initial presentation (confusion, blood lactate, creatinine, systolic blood pressure, sinus rhythm, and left ventricular ejection fraction).
