# Supplementary material for: Current real-life use of vasopressors and inotropes in cardiogenic shock - adrenaline use is associated with excess organ injury and mortality
Source: Crit Care. 2016 Jul 4;20:208. doi: 10.1186/s13054-016-1387-1 (PMC4931696; doi:10.1186/s13054-016-1387-1)
Supplement: Additional file 4: Table S3. — Baseline characteristics used in propensity score matching in relation to 90-day mortality in patients treated with vasopressors, and the balance achieved between the propensity-score-matched groups. (PDF 28 kb) [file 13054_2016_1387_MOESM4_ESM.pdf]

Table S2. Baseline characteristics used in propensity score matching in relation to 90-day mortality in patients treated with vasopressors and the balance achieved between the propensity score-matched groups.

	**Vasopressor-treated patients**			**Propensity score matched cohorts**					
	**Imputed cohorts pooled**			**Imputed cohort 1 (n 40:40)**	**Imputed cohort 2 (n 39:39)**	**Imputed cohort 3 (n 41:41)**	**Pooled (n=40:40)**		
	**Alive at 90 days (n=101)**	**Dead at 90 days (n=82)**	**p**	**SMD (avg |SMD| 0.037)**	**SMD (avg |SMD| 0.053)**	**SMD (avg |SMD| 0.039)**	**Adrenaline**	**Other vasopressors**	**|SMD| (avg |SMD| 0.033)**
Age (years)	64.1	70.4	<0.001	-0.008	0.099	0.027	68.3	67.9	0.044
Women (%)	21%	33%	0.06	0	0	0	73%	73%	0
Previous MI	15%	39%	<0.001	0.054	0	0	28%	27%	0.018
Previous CABG	1%	15%	<0.001	0	-0.090	0.086	9%	9%	0.059
History of hypertension	53%	68%	0.04	0.105	0.054	0.103	70%	65%	0.088
History of renal insufficiency	7%	20%	0.01	0	0.128	0	18%	20%	0.043
ACS etiology	78%	88%	0.09	0	0	0.072	87%	86%	0.024
Resuscitation prior to enrolment	24%	38%	0.04	0	0	0.049	37%	36%	0.016
Systolic BP (mmHg)	80	74	0.005	0.023	0.114	-0.012	77.7	77.1	0.050
Sinus rhythm	86%	68%	0.004	-0.055	-0.056	0.107	72%	75%	0.073
Confusion	59%	83%	<0.001	0.121	0	0	95%	94%	0.040
LVEF [%]	35	29	0.001	-0.053	0.112	-0.042	32	32	0.069
Creatinine (µmol/l)	108	156	<0.001	-0.004	-0.049	-0.029	144	147	0.027
Lactate (mmol/l)	3.1	6.4	<0.001	-0.125	-0.062	0.018	5.4	5.7	0.068
Propensity score	-	-	-	0.008	0.027	0.039	0.363	0.359	0.024

Results shown as % for categorical and means for continuous variables. Balance between the matched groups was assessed with standardized mean difference (SMD) of covariates and propensity score, and by the average of the absolute SMDs (avg |SMD|) of covariates.

MI = myocardial infarction, PCI = percutaneous coronary intervention, CABG = coronary artery bypass graft surgery, LVEF = left ventricular ejection fraction
