# Supplementary material for: Current real-life use of vasopressors and inotropes in cardiogenic shock - adrenaline use is associated with excess organ injury and mortality
Source: Crit Care. 2016 Jul 4;20:208. doi: 10.1186/s13054-016-1387-1 (PMC4931696; doi:10.1186/s13054-016-1387-1)
Supplement: Additional file 5: Figure S2. — Hemodynamics and plasma biomarkers in non-resuscitated patients receiving adrenaline or other vasopressor(s). (PDF 476 kb) [file 13054_2016_1387_MOESM5_ESM.pdf]

Figure S2. Hemodynamics and plasma biomarkers in non-resuscitated patients receiving adrenaline (dark grey; A) or other vasopressor(s) (light grey; O).

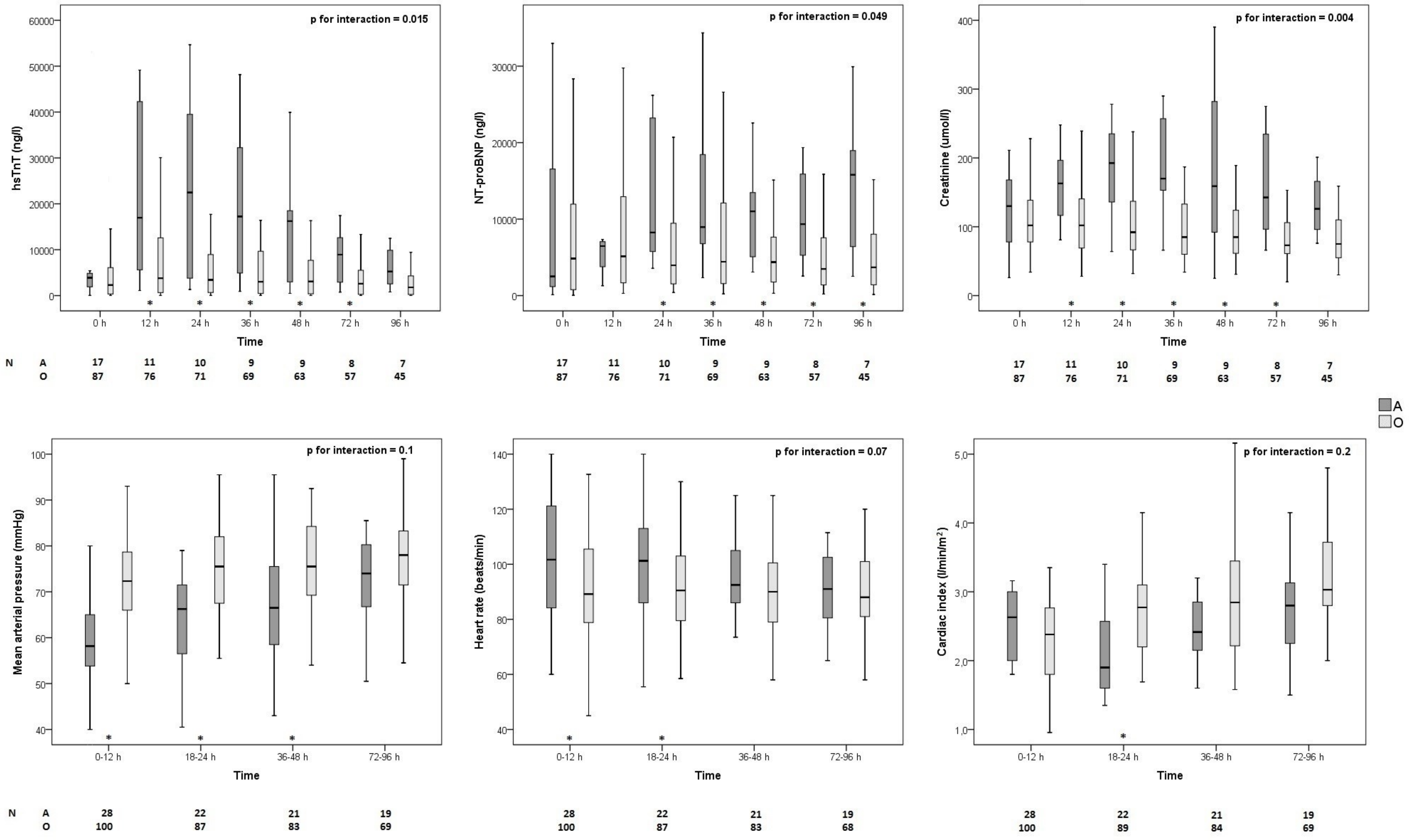

Figures represent box plots (central line = median, box = interquartile range, whiskers = minimum and maximum with outliers excluded) of separate measurements in each time point in the upper row (biomarkers) and the mean values of time intervals in the lower row (hemodynamics).

A = adrenaline, O = other vasopressor, hsTnT = high sensitivity troponin T, NT-proBNP = N-terminal pro-B-type natriuretic peptide

* = $p < 0.05$ for difference between adrenaline and other vasopressors

P for interaction = P value for time-by-group interaction
