# Supplementary material for: Current real-life use of vasopressors and inotropes in cardiogenic shock - adrenaline use is associated with excess organ injury and mortality
Source: Crit Care. 2016 Jul 4;20:208. doi: 10.1186/s13054-016-1387-1 (PMC4931696; doi:10.1186/s13054-016-1387-1)
Supplement: Additional file 6: Table S4. — Maximum infusion rates of vasoactive medications in patients treated with and without adrenaline. (PDF 8 kb) [file 13054_2016_1387_MOESM6_ESM.pdf]

Table S4. Maximum infusion rates of vasoactive medications in patients treated with and without adrenaline.

	Maximum infusion rate (ug/kg/min; median (IQR)		
	Patients treated with adrenaline	Patients treated without adrenaline	**p**
Noradrenaline	0.50 (0.34-1.06)	0.24 (0.13-0.43)	<0.001
Dopamine	8.3 (5.0-11.3)	5.0 (3.1-10.8)	0.3
Dobutamine	11.4 (6.7-24.4)	8.1 (6.0-14.1)	0.05
Levosimendan	0.13 (0.10-0.44)	0.14 (0.10-0.21)	0.5

IQR = interquartile range
