# Supplementary material for: Current real-life use of vasopressors and inotropes in cardiogenic shock - adrenaline use is associated with excess organ injury and mortality
Source: Crit Care. 2016 Jul 4;20:208. doi: 10.1186/s13054-016-1387-1 (PMC4931696; doi:10.1186/s13054-016-1387-1)
Supplement: Additional file 7: Figure S3. — Hemodynamics and biomarkers in patients receiving either a combination of noradrenaline and dobutamine or noradrenaline and levosimendan. (PDF 417 kb) [file 13054_2016_1387_MOESM7_ESM.pdf]

Figure S2. Hemodynamics and biomarkers in patients receiving either combination of noradrenaline-dobutamine (dark grey; N + D) or noradrenaline-levosimendan (light grey; N + L).

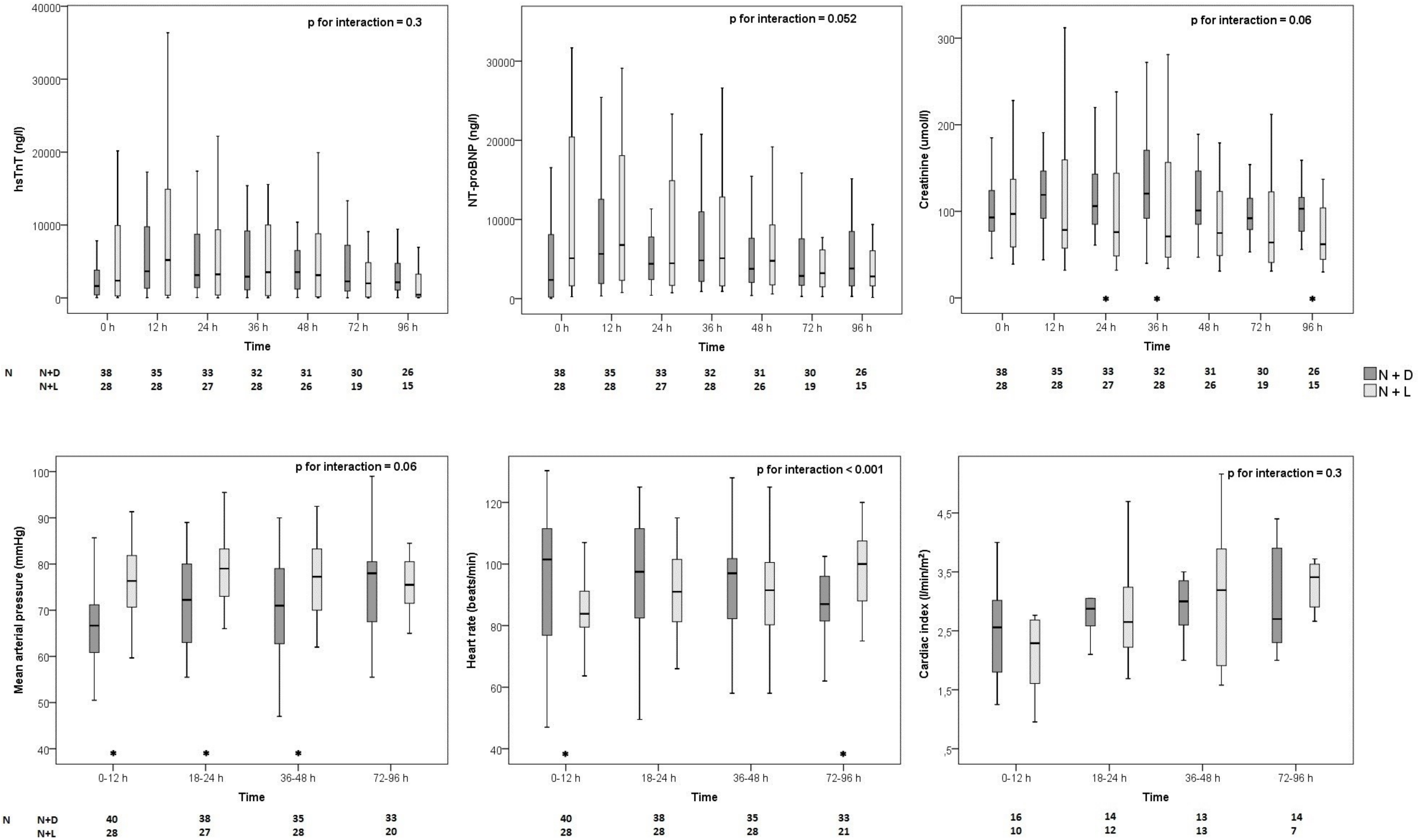
p for interaction = 0.3
hsTnT (ng/l)
Time
0 h 12 h 24 h 36 h 48 h 72 h 96 h
N N+D 38 35 33 32 31 30 26
N+L 28 28 27 28 26 19 15
p for interaction = 0.052
NT-proBNP (ng/l)
Time
38 35 33 32 31 30 26
28 28 27 28 26 19 15
p for interaction = 0.06
Creatinine (umol/l)
Time
38 35 33 32 31 30 26
28 28 27 28 26 19 15
N + D
N + L
p for interaction = 0.06
Mean arterial pressure (mmHg)
Time
0-12 h 18-24 h 36-48 h 72-96 h
N N+D 40 38 35 33
N+L 28 27 28 20
p for interaction < 0.001
Heart rate (beats/min)
Time
40 38 35 33
28 28 28 21
p for interaction = 0.3
Cardiac index (l/min/m²)
Time
16 14 13 14
10 12 13 7

Figures represent box plots (central line = median, box = interquartile range, whiskers = minimum and maximum with outliers excluded) of separate measurements in each time point in the upper row (biomarkers) and the mean values of time intervals in the lower row (hemodynamics). Patients receiving both dobutamine and levosimendan, or adrenaline were excluded.

hsTnT = high sensitivity troponin T, NT-proBNP = N-terminal pro-B-type natriuretic peptide

* = $p < 0.05$ for pairwise comparison between the groups

P for interaction = P value for time-by-group interaction
